# Role and mechanism of PIM family in the immune microenvironment of diffuse large B cell lymphoma

**DOI:** 10.1186/s12957-023-02947-5

**Published:** 2023-03-04

**Authors:** Changying Wang, Qitian Chen, Haichao Luo, Ran Chen

**Affiliations:** Department of Oncology, Xiangyang No.1 People’s Hospital, Hubei University of Medicine, Xiangyang, 441000 China

**Keywords:** PIM, DLBCL, Immune microenvironment

## Abstract

**Background:**

Diffuse large B cell lymphoma (DLBCL) is a more common non-Hodgkin lymphoma (NHL). This study aims to explore the prognostic value of PIM kinase family in DLBCL and its relationship with the immune microenvironment, to provide a certain reference for the prognosis and treatment of DLBCL.

**Methods:**

The prognostic value of PIM kinase family in DLBCL from the data set GSE10846 was verified through survival analysis and cox regression analysis. Mutations in PIM kinase family and its relationship with immune cell infiltration were explored with online cBioPortal, TIMER database, and single-gene GSEA analysis. Finally, the expression of PIM kinase family in tissues from DLBCL clinical samples was validated through immunohistochemical staining.

**Results:**

The proteins of PIM kinase family were highly expressed in DLBCL patients, which are good prognostic factors for DLBCL patients. Then, PIM1-3 proteins were positively correlated with the immune infiltration of B cells, whose types of mutations also showed different degrees of correlation with B cells. PIM kinase family proteins also showed a high correlation with PDL1. In addition, PIM kinase family was also associated with the commonly mutated genes in DLBCL, such as MYD88, MYC, and BTK.

**Conclusion:**

PIM kinase family may be a potential therapeutic target for DLBCL patients.

## Introduction

Diffuse large B-cell lymphoma (DLBCL) is the most common non-Hodgkin lymphoma (NHL), representing approximately 30 ~ 40% of newly diagnosed NHL patients. DLBCL is an invasive and clinically heterogeneous malignant lymphoma that grows rapidly and varies greatly in clinical efficacy, prognosis, and outcome [1]. The main treatment for DLBCL patients was once chemotherapy mainly based on CHOP regimen, which have a complete remission in 30 ~ 40% of the patients. Currently, the standard treatment for DLBCL patients is R-CHOP regimen, which adds rituximab (an antibody against CD20 human immunoglobulin) to the CHOP chemotherapy regimen. With the R-CHOP regimen, the cure rate of DLBCL patients reaches 75%, and the 5-year overall survival (OS) reaches 58%, which is significantly better than the treatment with the CHOP regimen alone [2]. However, the R-CHOP regimen is still ineffective for some patients, about 30 ~ 40% of which will relapse, which is also the main cause of death from this disease [3]. Therefore, it is urgent to find ways to effectively reduce the recurrence rate of this disease, prolong the survival of DLBCL patients, and improve patients’ outcomes as soon as possible.

In recent years, with the further study of the molecular pathways, targeted therapy, and immunotherapy of DLBCL, scholars have found that some genes and the expression of related proteins may play a key role in the occurrence and development of DLBCL. PIM kinases are over-expressed in a variety of malignancies, which play a key role in regulating the apoptosis, growth cycle, and proliferation of cells, so PIM kinases have become a popular therapeutic target in tumors at present [4]. PIM is a proto-oncogene encoding filament/threonine protein kinase and consists of three members, including PIM1, PIM2, and PIM3. Studies have demonstrated that PIM participates in the proliferation, differentiation, and apoptosis of cells, as well as the occurrence and development processes of tumors. PIM1 was found to be highly expressed in various tumors, including lymphoma, leukemia, bladder cancer, and prostate cancer [5]. There are NF-κB binding sites in PIM1 gene, and the activated NF-κB can increase the transcription of PIM1 gene, resulting in high expression levels of PIM1 proteins [6]. PIM1 can enhance the activity of Bcl-2 by phosphorylating B cell lymphoma-2 protein (Bcl-2), thus promoting the proliferation of tumor cells and further promoting the invasion and migration of tumors [7]. It was also found that PIM1 can also phosphorylate the pro-apoptotic protein Bad, inactivating it, preventing the apoptosis of tumor cells, and promoting the survival of the cells [8]. PIM2 is highly expressed in malignancies, such as lymphoma and multiple myeloma [9]. PIM2 functions similarly to PIM1, which seals Bad-induced apoptosis by phosphorylating Bad protein [10]. PIM2 can also activate the survival pathways of cells by increasing IκB kinase activity and activating the NF-κB signaling pathway to promote the survival of cells [11]. PIM3 is highly expressed in tumors, such as lymphoma, lung, gastric, liver, and pancreatic cancer [4]. It can promote the survival of cells and inhibit the apoptosis of cells by phosphorylating various proteins and genes, thus increasing the proliferation of normal cells or tumor cells [12]. It can also regulate the apoptosis by phosphorylating Bad protein, preventing Bad from binding to the anti-apoptotic protein Bcl-xl [13], as well as inhibiting the apoptosis by increasing Bcl-2 expression [14]. Finding biological indicators that can effectively predict efficacy or recurrence is beneficial for patients with poor prognosis to receive more aggressive treatments early. At present, there are very few studies on the relationship between PIM kinase family and the response to the treatments for DLBCL and its prognosis, and the relationship between them is still unclear.

In this study, GEO dataset (transcriptome dataset GSE10846), online cBioPortal, and TIMER database were comprehensively analyzed, to identify the relationship between PIM kinase family and the immune microenvironment for DLBCL. Interestingly, PIM kinase family was strongly associated with both B cells and T cells, while the immune cells are important agents for immunotherapy. Furthermore, PIM kinase family was significantly associated with DLBCL-mutated genes. In conclusion, PIM kinase family may be a potential target for the treatment of DLBCL patients.

## Methods and materials

### Acquisition of datasets

GSE10846 transcriptome dataset containing 420 DLBCL tissue samples and complete clinical information with follow-up information was downloaded from GEO (Gene Expression Omnibus) (https://www.ncbi.nlm.nih.gov/geo/) [15]. Also, 16 clinical paraffin tissue samples with DLBCL collected from the Department of Oncology of Xiangyang No.1 People’s Hospital, Hubei University of Medicine were included in this study. Among them, there were 8 cases of GCB and 8 cases of non-GCB, all of which were treated with large B and CHOP programs within the node. The IPI scores were 2–3 points, ranging from low-medium risk to medium–high risk.

### Survival analysis

The relationship between PIM family and the OS of DLBCL patients was determined through the Kaplan–Meier survival analysis with the “survival” and “survminer” packages in R language (www.r-project.org).

### cBioPortal online analysis

Gene mining and analysis were performed on the cBioPortal (https://www.cbioportal.org/), and the TCGA-DLBC database (Lymphoid Neoplasm Diffuse Large B-cell Lymphoma (TCGA, Firehose Legacy)) was selected for “exploring selected studies.” Then, PIM kinase family was analyzed to mine genes through the models of “Plots,” “Mutations,” “Cancer Types Summary,” and “OncoPrint.”

### Analysis of infiltration of immune cells

The correlation between mRNA expression of PIM kinase family and the infiltration levels of immune cells in lung cancer was assessed with the Pearson correlation coefficient. A previously published deconvolution method was used in TIMER to infer the abundance of tumor-infiltrating immune cells from gene expression profiles [16]. In TIMER, gene expression data, including 10,897 samples from 32 cancer types from the Cancer Genome Atlas (TCGA), were reanalyzed to estimate the abundance of six subpopulations of tumor-infiltrating immune cells (B cells, CD4T cells, CD8 T cells, macrophages, neutrophils, and dendritic cells) [17]. The expression of PIM kinase family in different tumors and the relationship between the expression of PIM kinase family and the abundance of immune infiltration were studied with the TIMER gene module. The data on the infiltration of B cells, CD8 + T cells, CD4 + T cells, dendritic cells, macrophages, and neutrophils are available from the TIMER database (https://cistrome.shinyapps.io/timer/).

### Single-gene GSEA analysis

The relationship between the expression of PIM kinase family in DLBCL and tumor signaling pathways was determined through GSEA (http://www.broadinstitute.org/gsea/index.jsp). Median based on individual gene expression was used for grouping. The correlation with the signaling pathway was verified according to the expression level of the gene. Statistical significance was defined with a normalized *P*-value of *P* < 0.05. A positive NES value indicated that the high expression of the gene was positively correlated with the signaling pathway, and vice versa.

### Immunohistochemical staining

After the section of DLBCL paraffin tissues, the samples were kept in an oven at 55 °C for 60 min. Dried tissue sections were dewaxed and rehydrated. The dewaxing process was as follows: dimethylbenzene for 10 min, dimethylbenzene for 10 min, absolute ethyl alcohol for 10 min, absolute ethyl alcohol for 10 min, 95% ethyl alcohol for 10 min, 95% ethyl alcohol for 10 min, 85% ethyl alcohol for 10 min, 70% ethyl alcohol for 10 min, and tap water. Antigens were recovered after heating with citrate buffer and 3% H2O2 at high pressure for 10 min, and then sealed with 5% normal goat serum (NGS, SAP-9100, ZSGB-BIO) for 1 min at room temperature, which was then incubated with NGS-diluted PIM kinase family antibodies (PIM1: ab54503, PIM2: ab107102, PIM3: ab198842, USA, Abcam) (dilution, 1:300; Proteintech Group Inc.) overnight at 4 °C 1 h later. NGS was taken as a negative control. The samples were incubated with biotin-labeled goat anti-mouse/rabbit immunoglobulin G (SAP-9100, ZSGB-BIO) for 15 min on the following day, which were, then, incubated and colored with diaminobidine solution, counterstained with hematoxylin for 2 min, and sealed with neutral glue. The expression levels of proteins of PIM kinase family were assessed by the product of the percentage of positive cells and staining intensity.

### Statistical analysis

All statistical analyses were performed with the R software (Version 4.1.0). Wilcoxon rank-sum test is a non-parametric test to detect the differences between the two groups. The Pearson correlation coefficient was used to estimate the correlation between the two gene expression levels. *P* < 0.05 means that the difference was statistically significant.

## Results

### DLBCL patients with high expression of PIM kinase family had a poor prognosis

**Fig. 1:**
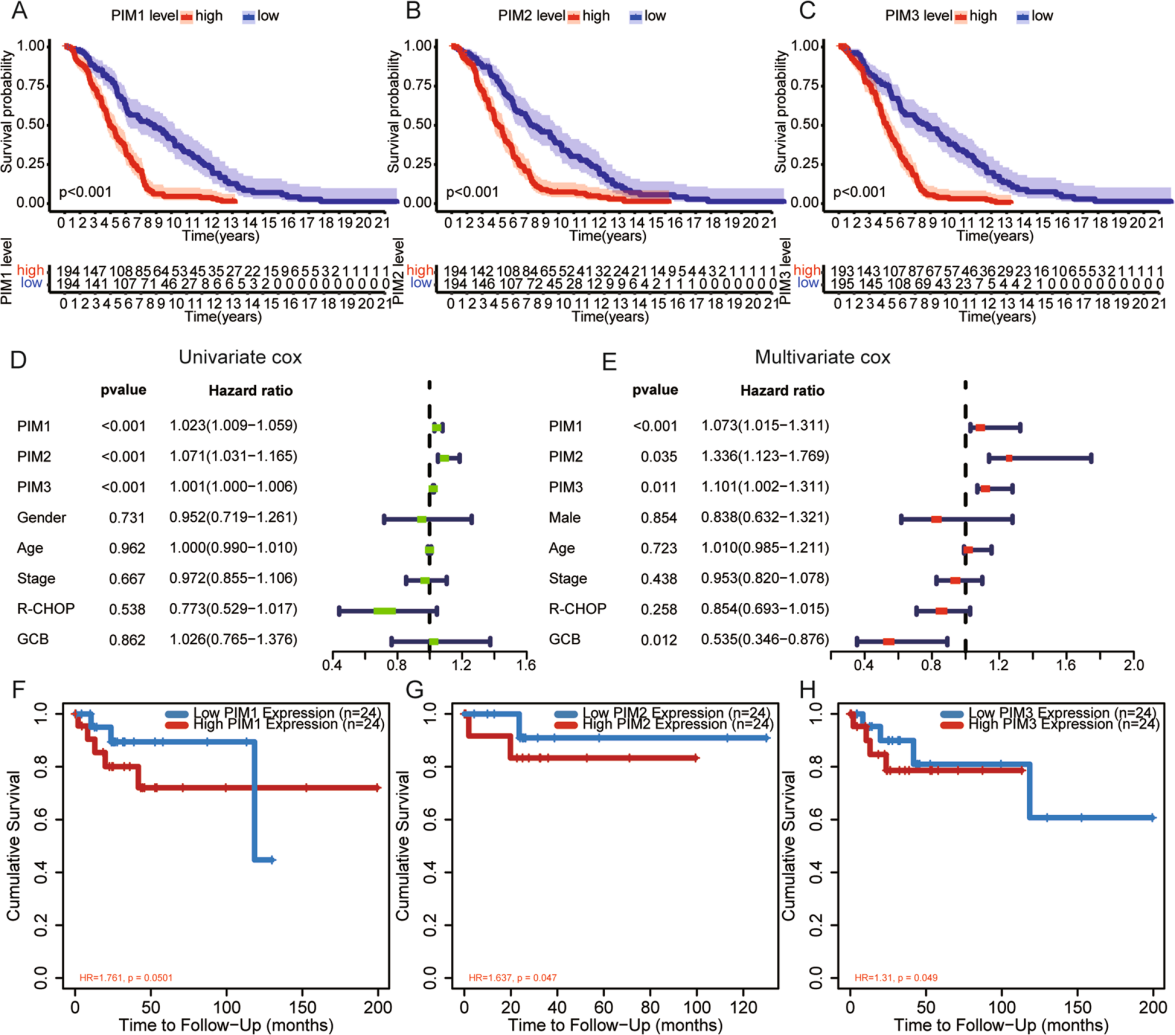
DLBCL patients with high expression of PIM kinase family have a poor prognosis. **A**–**C** Relationship between the expression level of PIM kinase family and OS of DLBCL patients. **D**, **E** Univariate and multivariate cox regression analyses to verify the independent prognostic value of PIM kinase family. **F**–**H** TIMER online database to visualize the relationship between the expression levels of PIM kinase family and OS of DLBCL patients

The prognostic value of PIM kinase family for DLBCL was verified with the dataset GSE10846. It was found that DLBCL patients with high expression of PIM1, PIM2, and PIM3 all had a poor OS (Fig. 1A–C). The results of univariate and multivariate cox regression analyses showed that PIM1, PIM2, and PIM3 were all independent prognostic factors in DLBCL patients (Fig. 1D, E, PIM1: HR = 1.078 (1.019–1.315), PIM2: HR = 1.236 (1.057–1.969), PIM3: HR = 1.021 (1.002–1.211)). Then, PIM kinase family was verified through the online TIMER database, and the results showed that DLBCL patients with high expression of PIM1, PIM2, and PIM3 also had a poor OS (Fig. 1F–H).

### Mutations of PIM kinase family in DLBCL

**Fig. 2:**
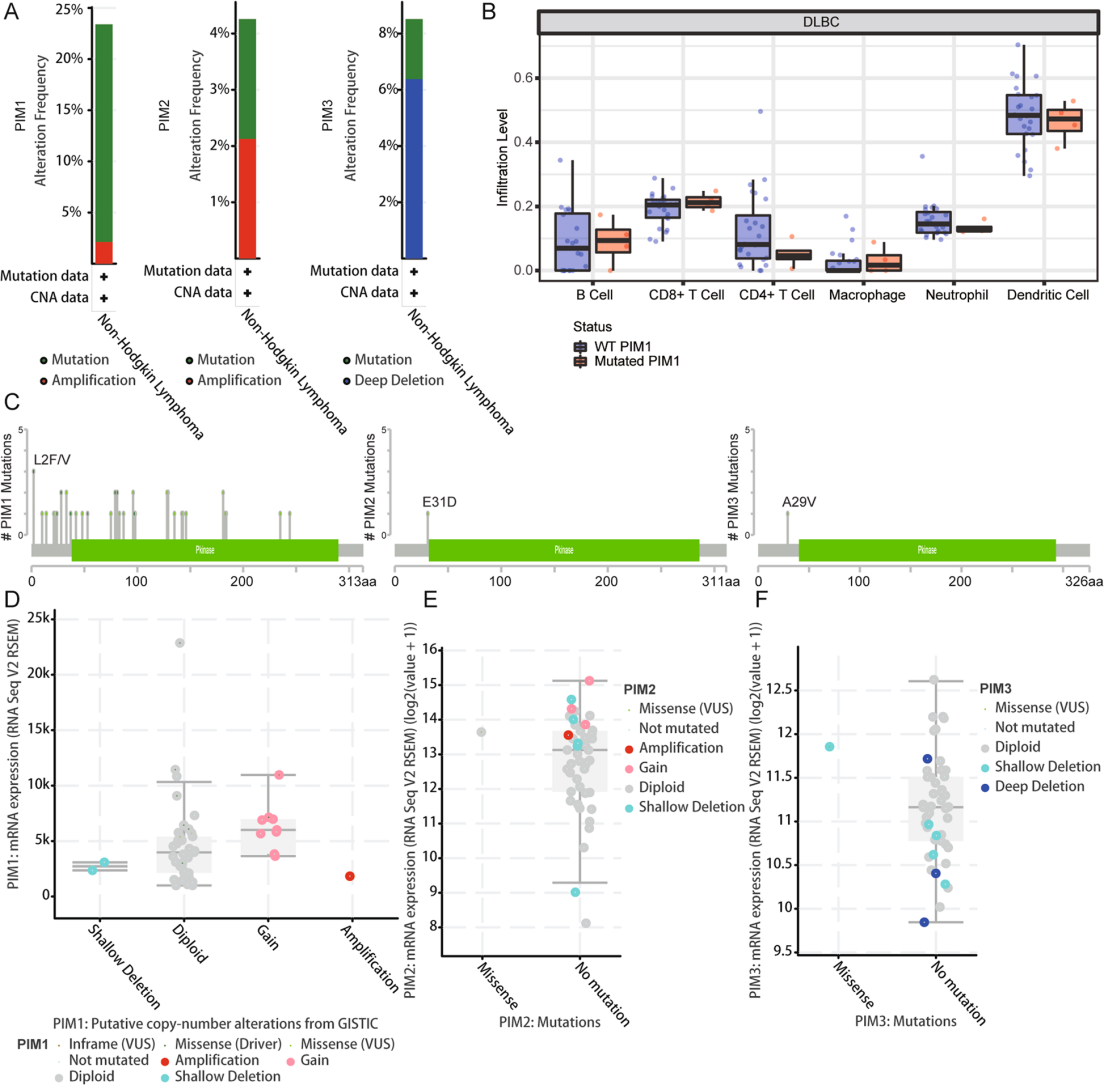
Mutations of PIM kinase family in DLBCL. **A** The mutation rates of PIM1, PIM2, and PIM3 were 23%, 4%, and 8%, respectively. **B** The relationship between the mutation status of PIM1 and the infiltration of immune cells. **C** The mutation sites of PIM1, PIM2, and PIM3 in DLBCL were L2F/V, e31d, and a29v, respectively. PIM1, PIM2, and PIM3 have mutations in DLBCL, which were L2F/V, E31D, and A29V, respectively. **D**, **E** Mutations in PIM1 and PIM2 included shallowdeletion, diploid, gain, and amplification. F Mutations in PIM3 included shallowdeletion and deepdeletion

According to the online cBioPortal analysis, the mutation rates of PIM1, PIM2, and PIM3 were 23%, 4%, and 8% in the TCGA dataset, respectively. PIM1 and PIM2 mainly showed mutation and amplification, while PIM3 mainly showed mutation and deepdeletion (Fig. 2A). Unfortunately, PIM1 mutations had little to do with immune cell infiltration (Fig. 2B). The mutation sites of PIM1, PIM2, and PIM3 in DLBCL were namely L2F/V, E31D, and A29V, respectively (Fig. 2C). In addition, the mutations in PIM1 and PIM2 appeared specifically as shallowdeletion, diploid, gain, and amplification (Fig. 2D, E). Mutations in PIM3 were specifically characterized as shallowdeletion and deepdeletion (Fig. 2F).

### Relationship between PIM kinase family and infiltration of immune cells

**Fig. 3:**
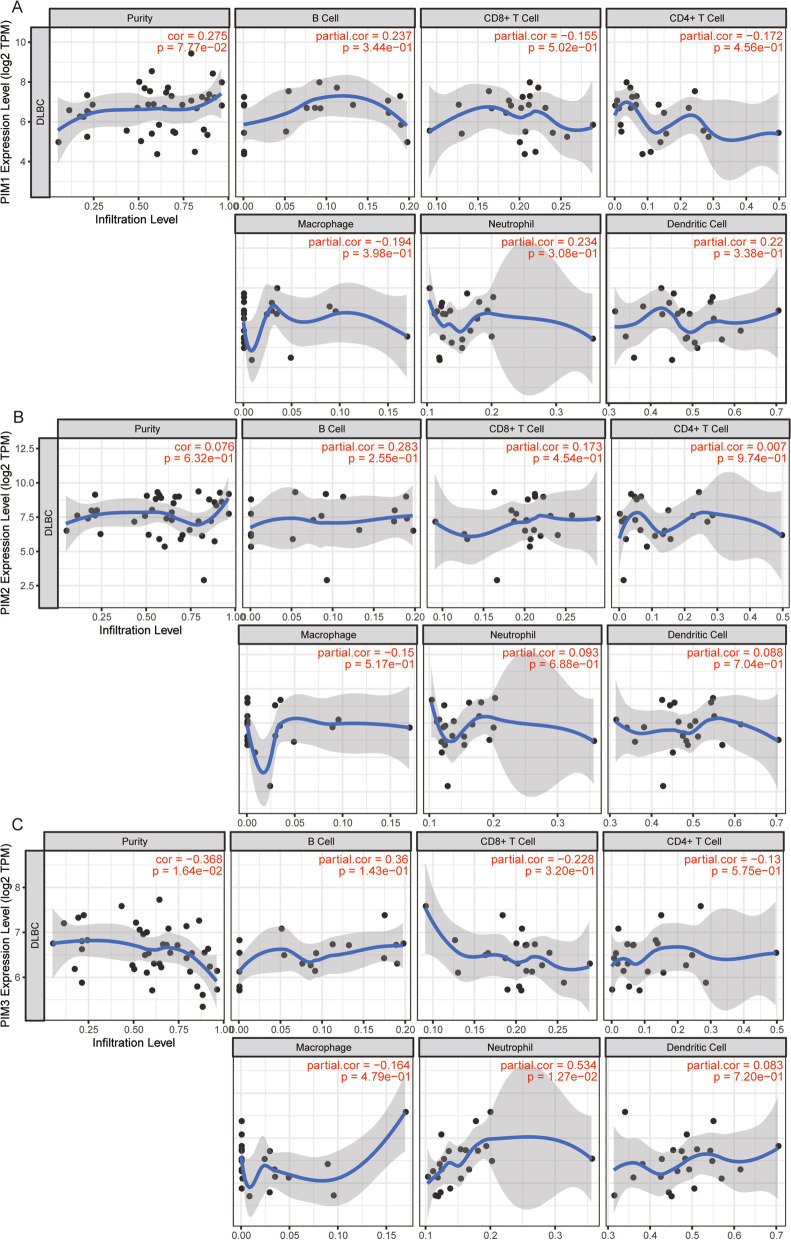
Relationship between PIM kinase family and the immune-infiltrating cells of DLBCL. **A** Correlation coefficients between PIM1 and B cells, CD8 + T cells, CD4 + T cells, macrophages, neutrophils, and DC cells, as well as mRNA expression of PIM1 were 0.237, − 0.155, − 0.172, − 0.194, 0.234, and 0.22, respectively. **B** Correlation coefficients between PIM1 and B cells, CD8 + T cells, CD4 + T cells, macrophages, neutrophils, and DC cells, as well as mRNA expression of PIM2 were 0.283, 0.173, 0.007, − 0.15, 0.093, and 0.088, respectively. **C** Correlation coefficients between PIM1 and B cells, CD8 + T cells, CD4 + T cells, macrophages, neutrophils, and DC cells, as well as mRNA expression of PIM3 were 0.36, − 0.228, − 0.13, − 0.164, 0.534, and 0.083, respectively

**Fig. 4:**
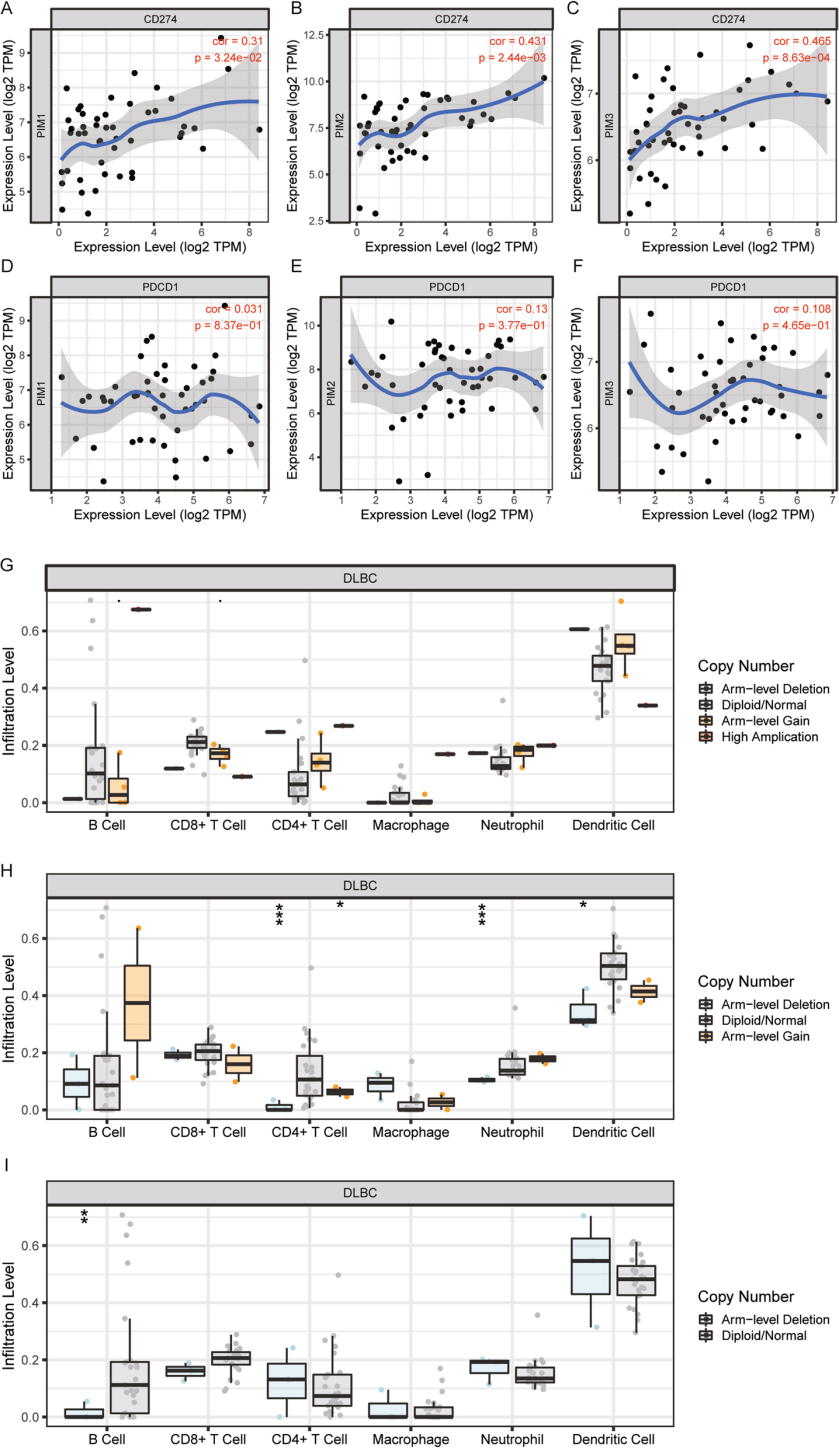
Relationship between the PIM kinase family and immunotherapy-related genes in DLBCL patients. **A**–F Pearson correlation analysis of the expression of PIM kinase family in DLBCL and the expression of PD-1 (PDCD1) and PD-L1 (CD274). **G**–**I** Correlation between mutation types of PIM kinase family and the infiltration of immune cells; the Arm-Level Gain mutation of PIM1 was associated with B cells and CD8 + T cells; the Arm-level Deletion mutation of PIM2 was associated with CD4 + T cells, neutrophils, and dendritic cells; the ARM-level Gain mutation of PIM2 was associated with CD4 + T cells; and the Arm-level Deletion mutation of PIM3 was associated with B cells

To further determine the relationship between PIM kinase family and immune function, an online TIMER database was performed for the infiltration analysis on immune cells. Correlation coefficients between PIM1 and B cells, CD8 + T cells, CD4 + T cells, macrophages, neutrophils, and DC cells, as well as mRNA expression of PIM1, were 0.237, − 0.155, − 0.172, − 0.194, 0.234, and 0.22, respectively (Fig. 3A). Correlation coefficients between PIM1 and B cells, CD8 + T cells, CD4 + T cells, macrophages, neutrophils, and DC cells, as well as mRNA expression of PIM2, were 0.283, 0.173, 0.007, − 0.15, 0.093, and 0.088, respectively (Fig. 3B). Correlation coefficients between PIM1 and B cells, CD8 + T cells, CD4 + T cells, macrophages, neutrophils, and DC cells, as well as mRNA expression of PIM3 were 0.36, − 0.228, − 0.13, − 0.164, 0.534, and 0.083, respectively (Fig. 3C). Moreover, PIM kinase family was positively associated with PD-L1 (CD274), but relatively less strongly associated with PD1 (PDCD1) (Fig. 4A–F). Interestingly, according to the specific types of mutations, the Arm-Level Gain mutation of PIM1 was associated with B cells and CD8 + T cells (Fig. 4G); the Arm-level Deletion mutation of PIM2 was associated with CD4 + T cells, neutrophils, and dendritic cells; the ARM-level Gain mutation of PIM2 was associated with CD4 + T cells (Fig. 4H); and the Arm-level Deletion mutation of PIM3 was associated with B cells (Fig. 4I).

### Relationship between PIM kinase family and DLBCL-mutated genes

**Fig. 5:**
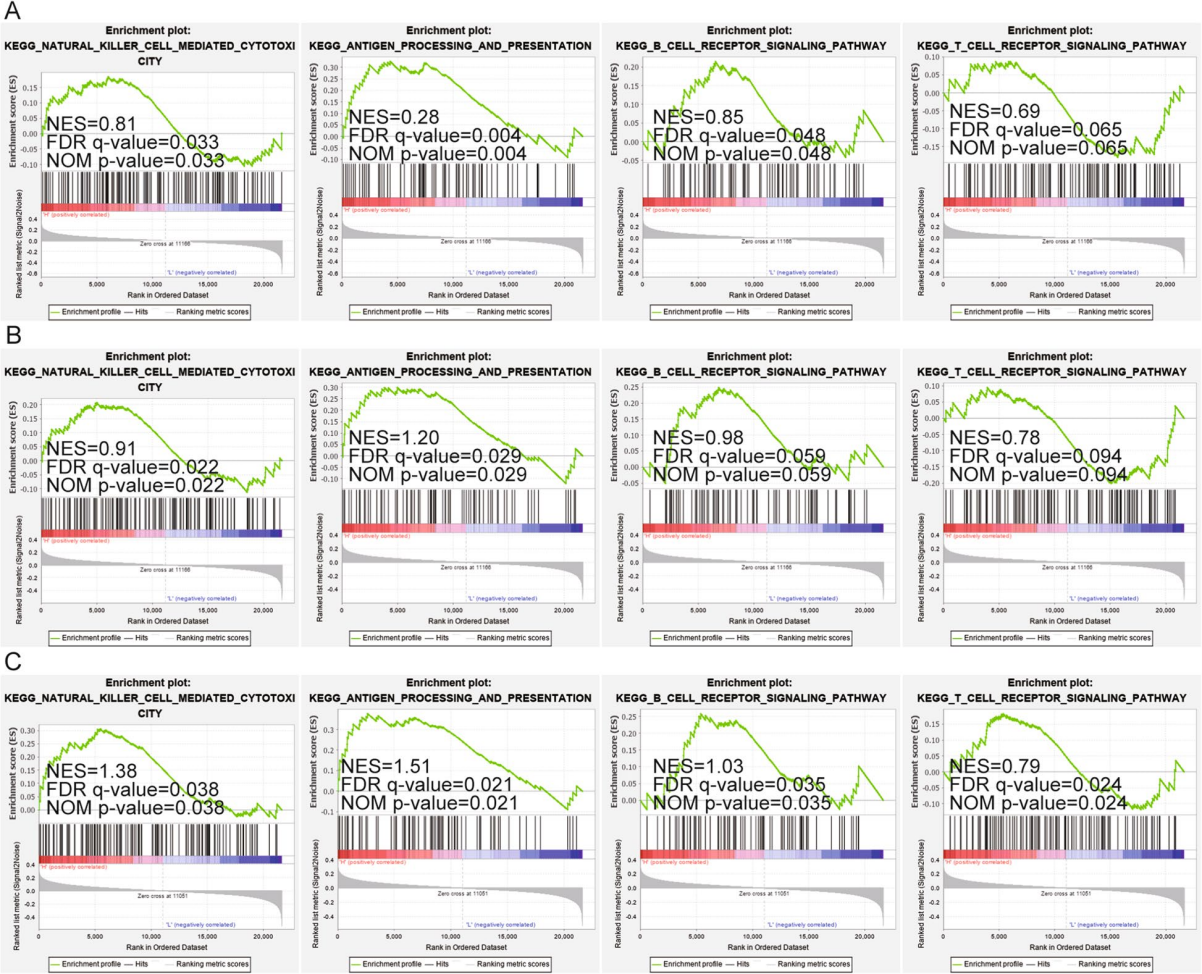
GSEA analysis on PIM kinase family in DLBCL. **A**–**C** Relationship of PIM kinase family with NK cell-mediated toxicity, antigen presentation, B cell receptor signaling pathway, and T cell receptor signaling pathway in DLBCL

**Fig. 6:**
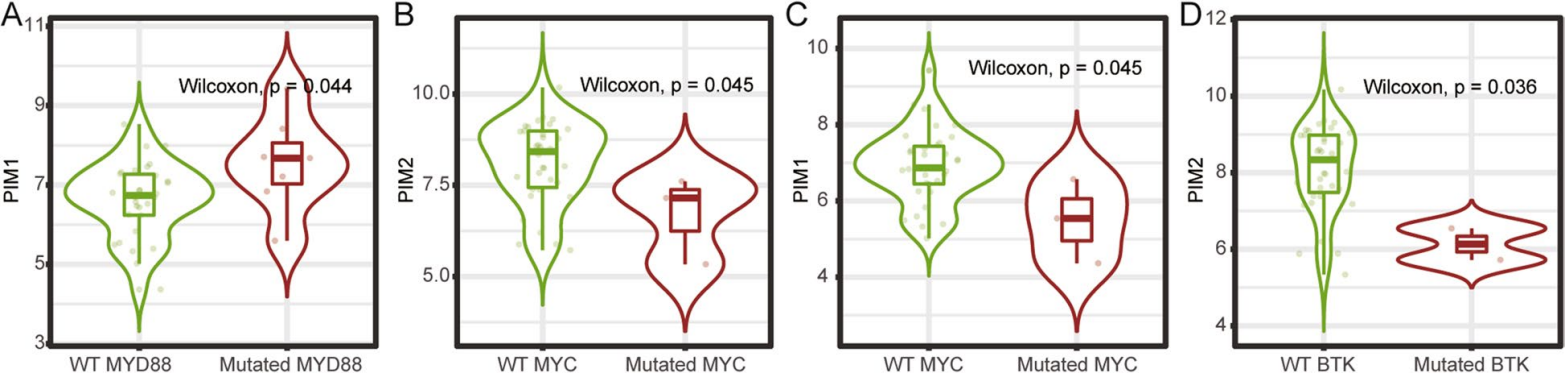
Relationship between PIM kinase family and DLBCL mutant genes. **A** PIM1 was positively correlated with MYD88 mutation. **B** PIM2 was negatively correlated with MYC mutation. **C** PIM1 was negatively correlated with MYC mutation. **D** PIM2 was inversely correlated with BTK mutation

To further determine the relationship between the expression of PIM kinase family and immune signaling pathways, PIM kinase family was studied through the single-gene GSEA analysis separately. PIM kinase family was mainly related and positively correlated with antigen process and presentation, while PIM3 was significantly and positively correlated with related signaling pathways of NK cells, B cells, and T cells (Fig. 5A–C). In addition, the relationship of PIM kinase family with DLBCL mutated genes was analyzed, and the results showed that PIM1 was positively associated with MYD88 mutation (Fig. 6A). PIM1 and PIM2 were inversely associated with MYC mutations (Fig. 6B, C). And PIM2 was inversely correlated with BTK mutation (Fig. 6D).

### Expression of PIM kinase family in DLBCL samples

**Fig. 7:**
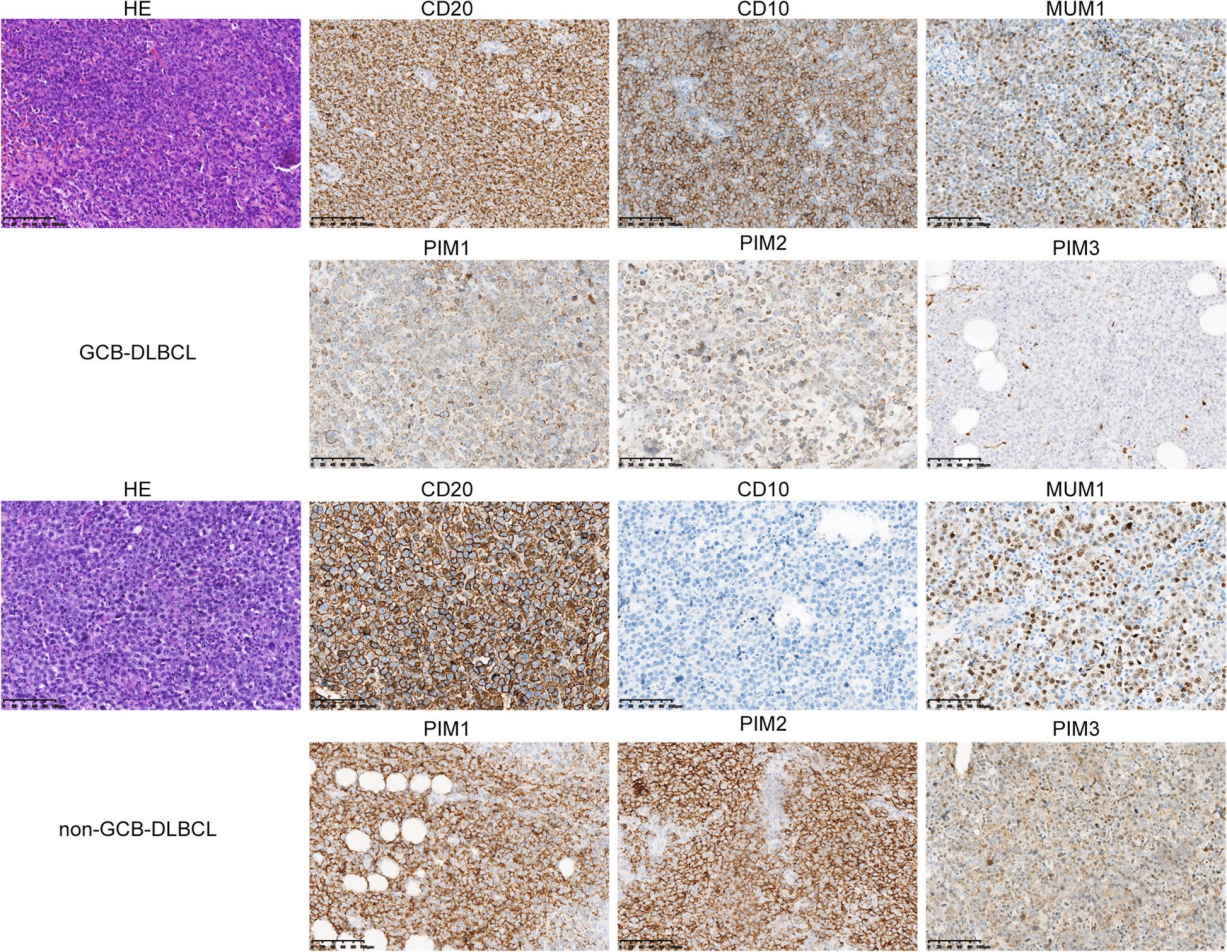
Expression of PIM kinase family in DLBCL samples. **A** Immunohistochemical staining of PIM family in DLBCL samples from GCB type. **B** Immunohistochemical staining of PIM family in DLBCL samples from Non-GCB type

Finally, the expression of PIM kinase family on the collected DLBCL clinical samples was verified. And it was found that all of the PIM family was expressed in DLBCL tissues. Interestingly, the expression of PIM kinase family in GCB DLBCL tissues were lower than those found in non-GCB DLBCL tissues. PIM1 and PIM2 were positive in both non-GCB and GCB. PIM3 was positive in non-GCB and negative in GCB. PIM1 and PIM2 were suspected to be strongly expressed in the cell membrane, and PIM3 was suspected to be strongly expressed in the cytoplasm (Fig. 7A, B).

## Discussion

DLBCL is an aggressive, rapidly growing, and clinically heterogeneous form of medium- to high-grade lymphoma, with multiple sub-types in WHO classification, and patients vary widely in various molecular genetic alterations, clinical efficacy, prognosis, and outcome. At present, the International Prognostic Index (IPI) widely used in the clinical assessment of prognosis cannot accurately judge the prognosis of many patients and cannot judge the prognosis of patients with the same IPI score. Although many prognostic indicators with DLBCL have been reported successively, none of the prognostic indicators related to DLBCL biology have been recognized [18]. With a deeper understanding of the pathogenesis of tumors, it is gradually found that some molecular biological indicators may play a key role in the development of tumors.

In this study, it was found that PIM kinase family proteins are highly expressed in DLBCL patients in the dataset GSE10846 and are poor prognostic factors for DLBCL patients. Genetic ablation of PIM kinase (PIM-1/2/3) or PIM inhibitor can induce apoptosis in DLBCL cell lines, providing new therapeutic hope targets for the disease [19]. Several studies have shown that PIM-1 expression can be found in various lymphomas, and its high expression is associated with a poor prognosis [20]. PIM447 (previously known as LGH447) is an orally available clinical pan-PIM kinase inhibitor, which is currently in the clinical evaluation on phase I of multiple myeloma [21]. Previous studies have reported a high expression of PIM-1 and PIM-2 in ABC-DLBCL, which helps to distinguish it from germinal center B cell subtype lymphoma [22]. At present, a large number of studies have shown that PIM-3 is mainly over-expressed in solid tumors, such as lung cancer, gastric cancer, pancreatic cancer, colorectal cancer, liver cancer, and prostate cancer. PIM-3 over-expression plays a certain role in the occurrence and development of tumors, and PIM-3 is even related to the prognosis and chemotherapy resistance of pancreatic cancer, liver cancer, and colorectal cancer [23–27].

Also, it was found that PIM1-3 proteins were positively associated with the immune infiltration of B cells, and their mutation types also showed different degrees of correlation with B cells. PIM1-3 proteins were over-expressed in malignancies of B cells, including chronic lymphocytic leukemia [28], Burkitt’s lymphoma [29], Chromosome 6-acquired non-Hodgkin’s lymphoma [30], and mantle cell lymphoma [31]. Among them, high expression levels of PIM1 and PIM2 were more common in DLBCL [19], which were associated with active STAT signaling, lymphoma proliferative activity, and higher disease stage. By means of immunohistochemical staining, it was found that the expression levels of PIM kinase family proteins can be found on tumor cells and microenvironment cells of DLBCL patients. Due to the growth mode of the diffuse infiltration of DLBCL, tumor cells are closely arranged with microenvironmental cells; therefore, it is difficult to distinguish between the two with immunohistochemical methods. PIM kinases also cooperate with other oncogenic genes associated with malignancies of B cells, such as c-Myc [19], nuclear factor kappaB [32], and CD40 [33], indicating that PIM kinase has a role in the genesis of malignancies of B cells. Furthermore, PIM kinase family was associated with the commonly mutated genes in DLBCL, such as MYD88, MYC, and BTK, which provides additional evidence that PIM kinase family may serve as targeted agents for DLBCL.

PD1, a member of the CD28/B7 family of immunoglobulin superfamilies, belongs to the immunosuppressive receptor. The main ligand of PD1 is PDL1 (also known as B7-H1), which is a type I transmembrane glycoprotein encoded by human CD274 gene [34]. When PD1 binds to PDL1, it suppresses the immune function of T cells. Preventing PD1 from binding to PDL1 can inhibit the PD1/PDL1-mediated immune escape of tumor cells and achieve the purpose of curing the tumor [35]. Currently, the aberrant expression of PD1 and PDL1 has been found in a variety of hematological malignancies [36]. Kwon et al. reported that PD-L1 had higher positive rates in some DLBCL patients with poor prognosis, regardless of tumor cells or microenvironmental cells [37]. Interestingly, a higher correlation of PIM kinase family proteins with PDL1 was also found, suggesting that PIM kinase family may have some potential in the immunotherapy for tumors.

The strength of this study are that it comprehensively analyzed the GEO dataset (transcriptome dataset GSE10846), online cBioPortal, and TIMER database to identify the relationship between PIM kinase family and the immune microenvironment of DLBCL. Meanwhile, this study also has the following shortcomings: (1) the biological function of PIM kinase family in DLBCL has not been further determined and (2) the expression of PIM kinase family in immune cells and its relationship to genetic mutations needs further design and verification.

## Conclusion

The findings identified the relationship of PIM kinase family with the prognosis and the immune microenvironment of DLBCL. PIM kinase family is strongly associated with B cells and T cells, and the immune cells are important agents of immunotherapy. Furthermore, PIM kinase family was significantly associated with DLBCL mutated genes. In conclusion, PIM kinase family may be a potential target for the treatment of DLBCL patients.

## Data Availability

The datasets used and/or analyzed during the current study are available from the corresponding author on reasonable request.
